# Ceruloplasmin: Macromolecular Assemblies with Iron-Containing Acute Phase Proteins

**DOI:** 10.1371/journal.pone.0067145

**Published:** 2013-07-03

**Authors:** Valeriya R. Samygina, Alexey V. Sokolov, Gleb Bourenkov, Maxim V. Petoukhov, Maria O. Pulina, Elena T. Zakharova, Vadim B. Vasilyev, Hans Bartunik, Dmitri I. Svergun

**Affiliations:** 1 Institute of Crystallography RAS, Moscow, Russia; 2 Structural Biology Unit, CICbioGUNE, Derio, Spain; 3 Institute of Experimental Medicine NWB RAMS, St.Petersburg, Russia; 4 EMBL Hamburg, Hamburg, Germany; 5 Research Unit for Structural Molecular Biology, Max-Planck Institute, Hamburg, Germany; University of Leeds, United Kingdom

## Abstract

Copper-containing ferroxidase ceruloplasmin (Cp) forms binary and ternary complexes with cationic proteins lactoferrin (Lf) and myeloperoxidase (Mpo) during inflammation. We present an X-ray crystal structure of a 2Cp-Mpo complex at 4.7 Å resolution. This structure allows one to identify major protein–protein interaction areas and provides an explanation for a competitive inhibition of Mpo by Cp and for the activation of *p*-phenylenediamine oxidation by Mpo. Small angle X-ray scattering was employed to construct low-resolution models of the Cp-Lf complex and, for the first time, of the ternary 2Cp-2Lf-Mpo complex in solution. The SAXS-based model of Cp-Lf supports the predicted 1∶1 stoichiometry of the complex and demonstrates that both lobes of Lf contact domains 1 and 6 of Cp. The 2Cp-2Lf-Mpo SAXS model reveals the absence of interaction between Mpo and Lf in the ternary complex, so Cp can serve as a mediator of protein interactions in complex architecture. Mpo protects antioxidant properties of Cp by isolating its sensitive loop from proteases. The latter is important for incorporation of Fe^3+^ into Lf, which activates ferroxidase activity of Cp and precludes oxidation of Cp substrates. Our models provide the structural basis for possible regulatory role of these complexes in preventing iron-induced oxidative damage.

## Introduction

Human ceruloplasmin (Cp) is a multi-functional copper-containing protein. Copper ions in the Cp molecule provide a large number of enzymatic activities. Indeed, Cp oxidizes highly toxic ferrous ions to the ferric state for further incorporation into *apo*-transferrins [1], catalyzes Cu(I) oxidation [2] and promotes the oxidation of biogenic amines (norepinephrine, serotonin) and synthetic amines (*p*-phenylenediamine (*p*-PD), *o*-dianisidine(*o*-DA)) [3]. Further, Cp is the only plasma protein demonstrating the activity of NO-oxidase, NO_2_^–^syntase [4], glutathione-linked peroxidase [5] and superoxide dismutase [6]. These properties make Cp an effective antioxidant, able to prevent oxidative damage to proteins, DNA and lipids [7].

Interactions of Cp with other proteins further widen the range of its functions. Protein-protein complexes are functional units of biological systems playing pivotal roles in most cellular processes, and the focus of modern structural biology is rapidly shifting towards the study of large macromolecular assemblies. Cp is suggested to participate in the regulation of clotting *via* its competition with blood coagulation factors FV and FVIII for protein C binding [8]. *In vitro* incorporation of Fe^3+^ into ferritin was shown to depend on formation of the Cp-ferritin complex [9]. The ability of Cp to interact with myeloperoxidase (Mpo) and to inhibit its pro-oxidant properties [10] likely imparts it with additional antioxidant activity *in vivo*. The interaction of Cp with neuropeptide PACAP 38 [11] probably plays a certain role in neuroregulation processes. Due to complex formation with ferroportin I, the membrane-bound Cp found in astrocytes participates in regulation of iron levels in the central nervous system and in the prevention of free radical reactions [12]. Cp may also be involved in regulating inflammation by interacting with macrophage migration inhibitory factor [13]. We have already described a complex of Cp with lactoferrin (Lf) [14]. In light of enhancement of Cp ferroxidase activity in the presence of Lf [15], the complex probably plays a role in iron metabolism. Recently we reported that, along with Mpo and Lf, some cationic proteins of leukocytes (*viz*.eosinophilic cationic protein, cathepsin G, neutrophilic elastase 3 and azurocidin) can be found complexed with Cp. It was supposed that the capability of Cp to form the complexes with these cationic proteins might be part of a regulatory mechanism in pathogenesis of systemic vasculitis [16].

Here we focus on the structural-functional features of interactions of Cp with Lf and Mpo.

All complexes described in the present study exist *in vivo*. The Cp-Lf complex is present in the colostrum of healthy women as a single component demonstrating ferroxidase activity [17]. Several different complexes of Cp with Lf and Mpo, including the ternary complex Cp-Lf-Mpo, were identified in samples of serum and purulent exudates from patients with various inflammatory diseases [18]. Autoantibodies against Mpo preventing Cp-Mpo interaction provoke the symptoms of systemic vasculitides [19]. Although the complex Cp-Mpo was not reported in the study, the results suggest that it may be important in the pathogenesis of this disease. Hypothesizing that interactions of Cp with Lf and Mpo may play important functional roles, we explored mutual influence of these proteins in complexes [20] and characterized the overall shape of Cp-Lf complex in solution using small angle X-ray scattering [21]. *Ab initio* structural analysis did not provide a unique SAXS model, but it revealed the stoichiometry of the complex and suggested the absence of major conformational rearrangements of either protein. These results were in good agreement with our previous biochemical studies which allowed us to select some sites in Cp likely involved in the interactions with Lf [14], [18]. However, structural details of the formation of the Cp-Lf, Cp-Mpo and Cp-Lf-Mpo complexes remained unclear.

Crystal structures of each of the three individual proteins i.e. Cp, Lf, Mpo have been determined. The Cp molecule (∼132 kDa) consists of a single polypeptide chain with 1046 amino acid residues. Its crystal structure obtained at 2.8 Å resolution distinguishes six ß-barrel homologous domains connected by flexible loops [22]. Six tightly bound copper ions, which can be divided into three types according to their spectral characteristics, were distributed irregularly among these six domains (**Figure S1A**). Domains 2, 4, and 6 contained one type I copper each. Three copper ions (two type III and one type II) form a trinuclear cluster with ligands provided by domains 1 and 6. Lf is a multifunctional glycoprotein (∼78 kDa) abundant in biological fluids and tissues. Lf maintains iron homeostasis, possesses wide-range antimicrobial activity and immunomodulatory properties, and is able to act as antitumor agent and transcriptional factor [23]. Lf is composed of two highly homological sequences known as N- and C-lobes. Each lobe contains one specific metal-binding site in a deep cleft between two dissimilar domains. The entire molecule thus comprises four domains, N1 and N2 in the N-lobe, and C1 and C2 in the C-lobe. The N1 and C2 are “external” while N2 and C1 are “internal” domains [24] (**Figure S1B**).

Mpo (EC 1.11.1.7) is one of the major proteins of the antimicrobial system of mammalian neutrophils [25]. The antipathogenic activity of Mpo is due to its ability to catalyze the two-electron oxidation of halide ions and thiocyanate to hypohalous acids and hypothiocyanate, which are effective antimicrobial agents [26]. Reactive species produced by the catalytic activity of Mpo are also thought to contribute to tissue damage associated with certain inflammatory diseases [27].

According to a 1.95 Å crystal structure [28], Mpo is a homodimer of 140 kDa, each monomer consisting of two polypeptides of 108 a.a. (light chain) and 466 a.a. (heavy chain) and containing a heme.

Despite the availability of individual crystal structures of Cp, Lf and Mpo, structural characterization of the binary and ternary complexes of these proteins was proven to be difficult in view of the size and lability of the proteins, especially of Cp and Mpo. Another complication arose due to the specific multidomain structure of Cp that contained many long flexible loops on the surface, which were extremely sensitive to proteolysis. The intrinsic flexibility of protein complexes and their large size often preclude high resolution crystallographic studies. The threshold of “acceptable” resolution for obtaining mechanistic insights is being pushed by recent structures at 3.8 to 4.7 Å [29]. The low resolution structural studies in solution using SAXS provide a useful alternative to low resolution X-ray crystallography, especially in case of crystallization problems. Moreover, SAXS can provide an independent evidence of stoichiometry, physiological states and quaternary structure of complexes in solution, complementing crystallographic data. Here we describe for the first time a low-resolution X-ray crystallographic structure of the Cp-Mpo complex and present SAXS-based structural models of Cp-Mpo, Cp-Lf and Cp-Lf-Mpo in solution. A re-refined structure of Cp at 2.6 Å [30] was employed for construction of models, elucidation of additional details of the structural organization of Cp, and for comparative analysis. The obtained structural models of the Cp complexes were in agreement with previous results on spectral and enzymatic properties. The models further revealed the areas of protein-protein interactions providing structural insights into the influence of the complex formation on the functions of individual proteins.

## Results

### Crystal Structure of Cp-Mpo and Cp with Free Labile Sites

**Figure 1:**
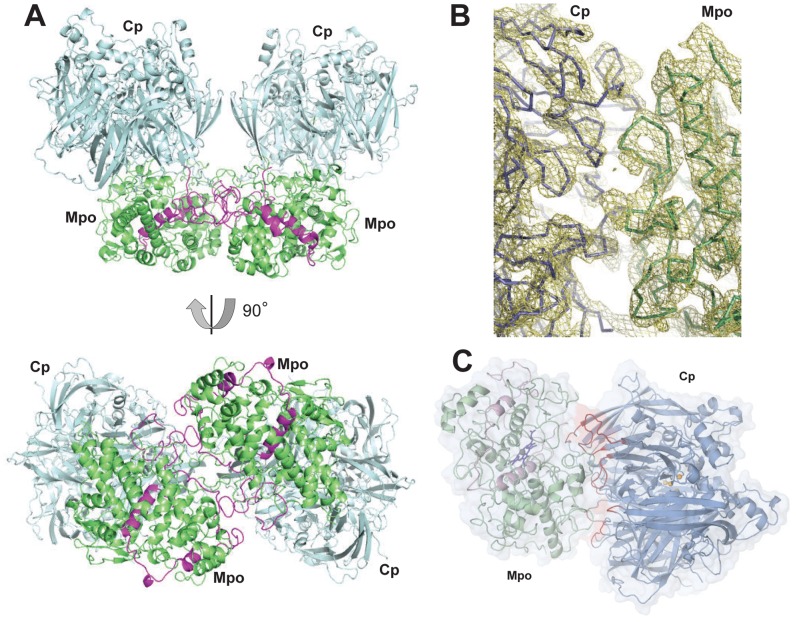
Structure of Cp-Mpo complex. (**A**) Symmetrical molecule of the complex given in cartoon representation. Cp is shown in palecyan, Mpo is shown in green (heavy chain) and magenta (light chain). Bottom view is rotated by 90 degrees around horizontal axis. (**B**) Electron density of a fragment of Cp-Mpo complex at 1σ level (golden mesh). Cp and Mpo backbones are shown in blue and green, respectively). (**C**) Cp-Mpo complex in cartoon and semi-transparent surface representation. Cp is shown in blue, Mpo is shown in green. Loops of Cp are involved in protein-protein interaction are shown in red.

The crystal structure of the Cp-Mpo complex was solved from the X-ray diffraction data at a resolution of 4.7 Å (**Table 1**). The crystallographic model revealed a complex stoichiometry of 2Cp:1 Mpo (**Figure 1A**). The independent part of the unit cell contained one of the two subunits of the dimeric Mpo and one Cp molecule. Our structure of Cp-Mpo was the first structure where we can see one monomer of Mpo in the asymmetric part (in all known human myeloperoxidase structures the asymmetric part contains a dimer [31]. It can be concluded that the monomers of naturally dimeric Mpo were crystallographically equal. In our case the overall structure of the Mpo monomer in the complex was similar to the crystal structures of the individual Mpo (e.g. pdb entries **1dnu, 3f9p**). Sugar chains between the Mpo monomers (which were symmetrical in our structure) were visible in the electron density map (**Figure S2 AB**). The two molecules of Cp in the Cp-Mpo complex were related by a 2-fold axis. The contacts between the two Cps within 3.5 Å were formed by the residues belonging to the loop 831–836 of one molecule and to the residues belonging to the loop 769–776 of the other molecule (**Figue S2 CD**).

**Table 1:** Data collection statistics and refinement for Cp-Mpo complex and Cp with free labile sites.

	Cp	Cp-Mpo
**Data collection**		
Space group	P3_2_21	P6_5_22
Cell dimensions		
*a,b,c* (Å)	210.78,210.78,84,50	106.3,106.3,836.5
α,β,γ (°)	90,90,120	90,90,120
Resolution (Å)	50-2.6(2.7-2.6)*	100-4.7(4.78-4.7)
R_merge_	4.5(30.5)	23.4(41.9)
I/σI	17.7(1.75)	2.8(2.8)
Completeness (%)	95.2(92.9)	97.1(99.7)
Redundancy	4.2(2.7)	5.1(6.4)
No. reflections	59625	15612(765)
**Refinement statistic**		
Resolution (Å)	15-2.6(2.7-2.6)	15-4.7(4.8-4.7)
*R*_work_/*R*_free_	19.9/23.4	36.5/40.0
No. atoms		
Protein	8399	12995
Ligand/ion	35	117
Water	93	–
*B*-factors		
Protein	27.99	–
Ligand/ion	39.5	–
Water	35.9	–
R.m.s. deviation		
Bond lengths (Å)	0.017	0.007
Bond angles (°)	1.673	1.241

* Values in parentheses are for highest resolution shell.

The secondary structure elements of Cp and Mpo were clearly recognizable in most parts of both molecules as expected for a 4.7 Å-resolution crystallographic map (**Figure 1B**).

However, it was not possible to unambiguously trace the majority of interdomain linking regions most likely because of their inherent flexibility (these linkers have thus not been included in the present model).

**Figure 2:**
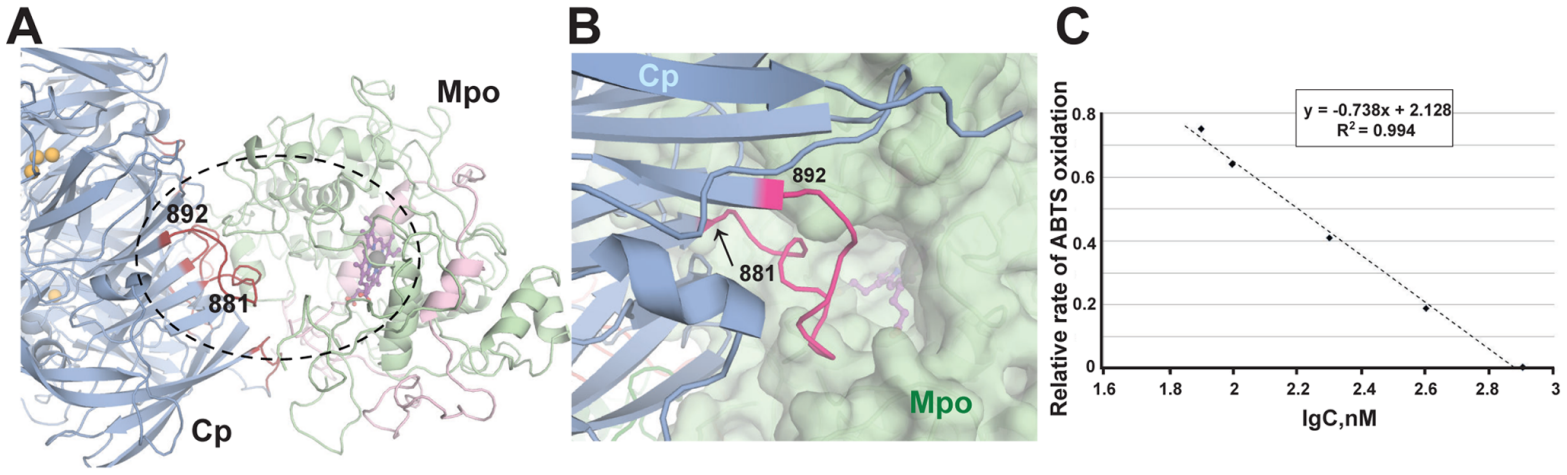
Interaction of extended loop 882-892 of Cp with Mpo. Cp is shown in blue, heavy chain of Mpo in palegreen, light chain of Mpo in pink. Copper ions are shown as orange spheres. Heme is shown in violet, ferrous is shown in gray sphere. (**A**) Overall view of Cp monomer contact with Mpo monomer. Selected contact is indicated by dash-line oval (**B**). Zoom of contact area of Cp with the entrance of heme pocket of Mpo. Cp is shown in cartoon; Mpo is shown in cartoon and semi-transparent surface representation. Ends of loop between domain 5 and 6 are marked. **(C)** Inhibition of ABTS-peroxidase activity of Mpo by synthetic peptide RPYLKVFNPR corresponding to the 883-892 fragment of Cp sequence.

The contact areas on the two proteins’ surfaces are shown in **Figure 1C** and **Figure 2A,B**. A yield of total contact surface area in Cp-Mpo interaction equals to 2400 Å^2^, which indicates a strong binding of the partners in the assembly [**32**]. Cp and Mpo have several regions of interaction.

When the complex is formed, the entrance into the Mpo heme pocket is likely to become occluded by the loop of Cp linking domains 5 and 6 (a.a. 885–892) (**Figure 2A,B**). This loop was never seen completely in all known Cp structures and was build using I-TASSER server [**33**].

A synthetic peptide RPYLKVFNPR corresponding to the fragment of Cp sequence 883–892 inhibits peroxidase activity of Mpo towards ABTS in a dose-depended manner (IC_50_∼160 nM) (**Figure 2C**). Moreover, the inhibitory effect of this peptide was comparable to that of Cp (K_i_ 0,1–1 µM), which further corroborated our suggestion about the contact of this fragment of Cp with the substrate binding site of Mpo [20].

**Figure 3:**
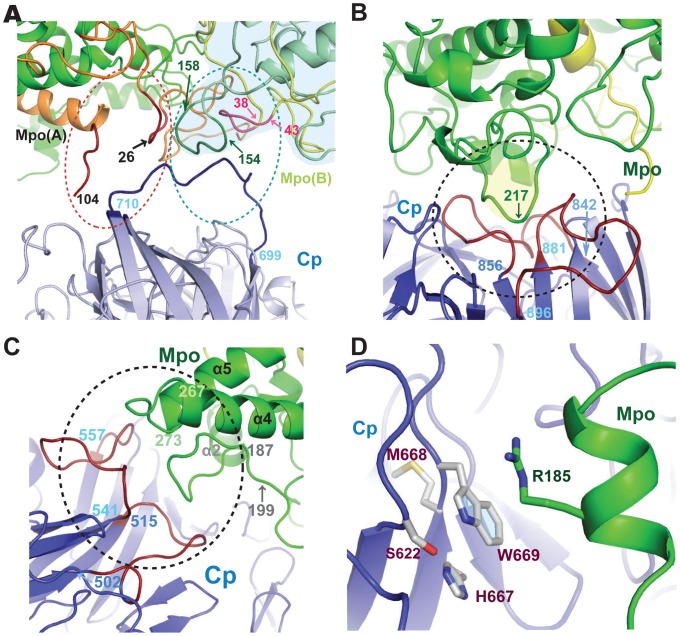
Contact areas of Cp with Mpo near 3,4 and 5 domains of Cp. (**A**) Contact area near N-terminal of Mpo light-chain and loop 699-720 of Cp. (**B**) Contact of 699-720 loop of Cp with symmetrical monomer of Mpo. Symmetrical molecule of Cp is not shown for clearance. (**C**) Contact area near 542-557 and 618-624 loops of Cp. (**D**) Contact area near *p-*PD site of Cp (domain 4). Residues M668, W669 and H667 are shown in stick representation.

It seemed likely that the extended loop of Cp (a.a. 699–710) can also be involved in the interaction with the N-terminal residues (1–27) of the light chain of Mpo (**Figure 3A**). This 699–710 loop also interacts with the symmetric monomer of Mpo, too (**Figure 3B**), thus establishing contacts with the Mpo dimer. The loops of the heavy and light chains of the second Mpo monomer were participating in these contacts.

The residues 191–193, 229, 272–274 of Mpo contact the Cp residues belonging to the domain 4 (a.a. 511, 542, 553, 554) and domain 6 (a.a. 989) (**Figure 3C**).

Another binding site was located in domain 4 of Cp near the side chain of Trp669. Arg185 of Mpo, located nearby (**Figure 3D**) was likely involved in the contact. Trp669 belongs to the *p*-PD binding site, formed also by Met668 and His667 [34]. The structural analysis of Cp with free labile sites reveals that Ser622 can belong to this binding site, too. Despite the medium resolution, it was possible to determine two alternative positions of His667 (**Figure S3**). We found a new possible conformation of H667, where this residue was turned outwards Trp669 and displayed a water-bridge with the side chain of Glu552 (in the conformation reported before, [34] His667 was stacked with Trp669). The side chain of Ser622 formed an additional H-bond with the side chain of His667 in this conformation, thus stabilizing the latter (**Figure S3**). A binding of the substrate could be a signal to the reorientation of His667.

### The Study of Ternary Cp-Lf-Mpo and Binary Cp-Lf Complexes by SAXS

**Figure 4:**
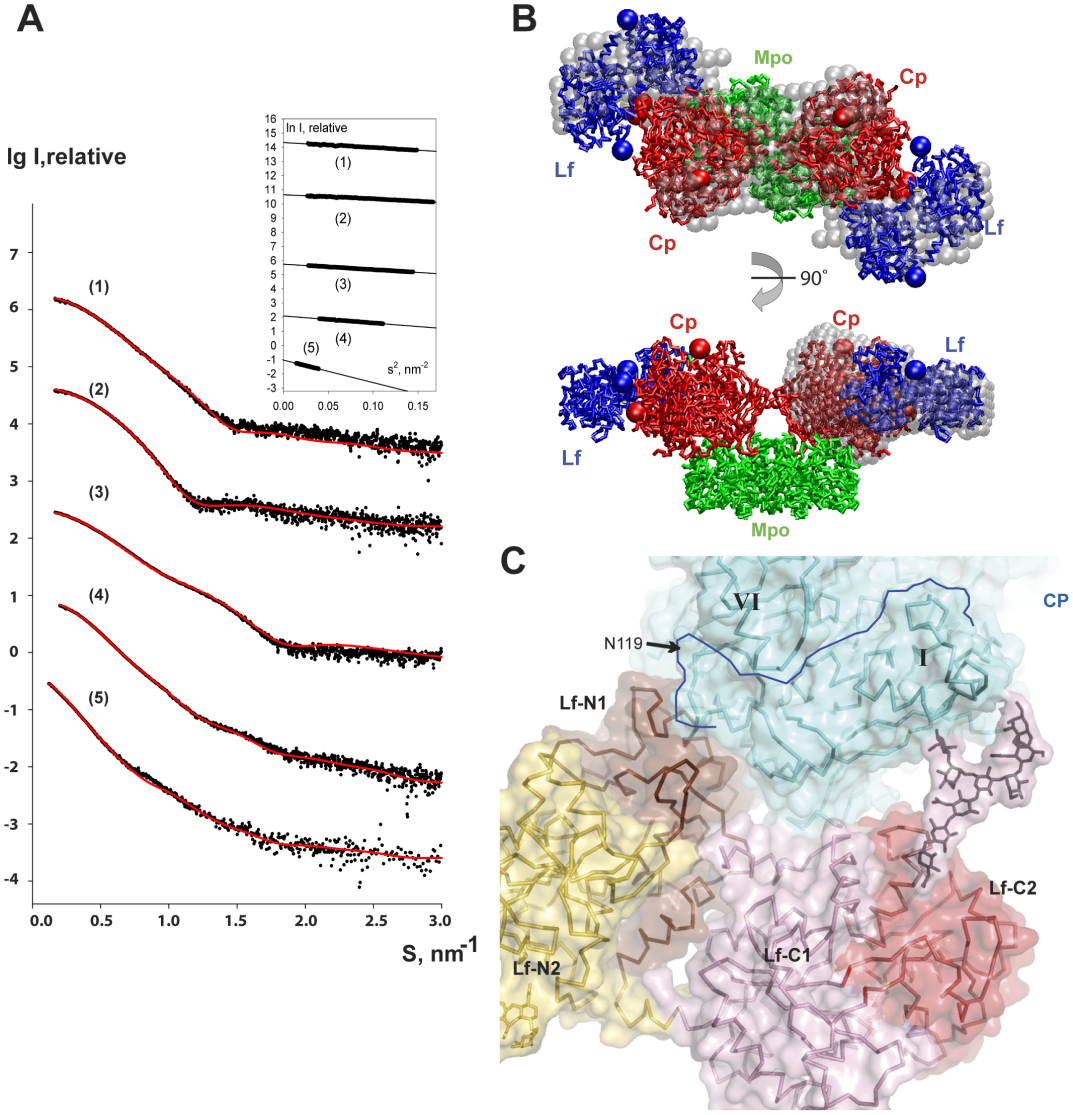
Cp-Mpo-Lf in solution. (**A**) Scattering patterns of ternary complex and individual proteins. (1): Mpo dimer, (2) Cp with glycans, (3) glycosylated Lf, (4) Monomeric binary complex Cp-Lf and (5) ternary complex. Experimental data are denoted by dots, fits computed from the appropriate portions of the SAXS model are shown as red solid lines. The plots are displaced along the logarithmic scale for better visualization. Insert: Guinier plots (ln I *versus* s^2^) with the linear fits (straight lines) in the ranges automatically determined by AutoRg (Petoukhov et al., 2012). (**B**) SAXS models of the ternary complex. Rigid body model is presented by Ca trace whereby Cp and Lf subunits (two copies each) and Mpo dimer are shown in red, blue and green, respectively. The location of sugars is denoted by appropriately colored solid spheres. Bottom view is rotated by 90 degrees around horizontal axis. *Ab initio* shapes of the ternary complex (top) reconstructed in P2 and of Cp-Lf (bottom) are shown as grey beads. (**C**) Zoom of Cp-Lf interaction in ribbon and transparent surface representation. One of possible Lf sugar chain conformation (brown sticks and pink semitransparent surface) is shown in stick and surface representation. Lf-N1 is shown in chocolate, Lf-N2 in yellow, Lf-C1 in pink and Lf-C2 in red. Cp is shown in light cyan. Border of domain 1 is highlighted by blue line. Glycosylation site N119 indicates by black arrow.

Solution scattering has been employed for the structural characterization of the ternary complex Cp-Lf-Mpo and of the binary construct Cp-Lf, for which no crystals were obtained. SAXS data was also collected for the three individual proteins i.e. Mpo, Cp and Lf, to help in the modeling of their glycosylation. The experimental scattering patterns from the measured constructs are presented in **Figure 4A** and the overall structural parameters computed from the SAXS data are given in Table 2. The linearity of the Guinier plots (insert in **Figure 4A**) suggested that all the constructs were monodisperse in solution. The obtained values indicated that Mpo, as expected, formed dimers in solution, whereas Cp and Lf were in monomeric states and their equimolar mixture yields a 1∶1 binary complex Cp-Lf. The increase in the excluded volume *V_p_* for Cp-Lf-Mpo indicated the binding of two Cp-Lf copies to an Mpo dimer. This was consistent with the present crystallographic model of Cp-Mpo and with the 2∶2:1 stoichiometry of the ternary complex Cp-Lf-Mpo proposed earlier in our study [35]. The significantly higher values of *R_g_* and *D_max_* for the ternary complex compared to those from the crystal structure of Mpo-Cp further suggested that Lf was located at the periphery of the complex.

**Table 2:** Overall parameters obtained by SAXS.

	R_g_, nm	D_max_, nm	V_p_, nm^3^	MM_est_, kDa	MM_mon_, kDa	Oligomericstate	?
**Mpo**	3.4±0.1	11.0±0.5	180±15	115±10	65	2	1.81
**Cp**	3.2±0.1	10.0±0.5	200±15	125±10	120	1	1.62
**Lf**	3.4±0.1	11.5±0.5	130±10	80±7	80	1	2.29
**CpLf**	4.0±0.1	12.5±0.5	320±20	200±15	200	1∶1	1.76
**CpLfMpo**	6.9±0.2	25.0±1.0	790±60	490±40	265	2∶2:2	1.74

R_g_, D_max_ and V_p_ are, respectively, radius of gyration, maximum size and excluded particle volume obtained from experimental SAXS profiles. MM_est_ is the molecular mass estimated from the excluded volume (using empiric factor of 0.625) and MM_mon_ is the molecular mass expected from monomeric sequence (or equimolar monomeric assemblies in case of binary and ternary complexes) with glycosylation taken into account. Oligomeric state is defined by the ratio of MM_est_ to MM_mon_. χ is discrepancy between the experimental SAXS data and the curve computed by CRYSOL from the rigid body model of corresponding construct with tentatively added carbohydrates.

Low resolution *ab initio* models were constructed for Cp-Lf and the ternary complex (**Figure 4B**), using the bead modeling program DAMMIN [36] and yielded good fits to the experimental data. The ternary complex reconstructions that were made utilizing no symmetry/two-fold symmetry constraints produced very similar models. The assumption of the P2 symmetry did not impair the quality of the fit, thus indicating that the ternary complex can possess the two-fold axis (as does the crystallographic model of Cp-Mpo). The volumes of the models for Cp-Lf and Cp-Lf-Mpo were in agreement with those computed directly from the SAXS data and they further supported the proposed stoichiometries of the complexes. The bead model of the ternary complex had an elongated shape and the crystal structure of Cp-Mpo overlapped well with the middle portion of the *ab initio* model. This suggested the same mutual arrangement of Mpo and Cp in the binary and the ternary complexes. The protrusions at the periphery of the *ab initio* model were able to accommodate both Lf subunits and thus confirmed their peripheral location.

The structures of the binary and the ternary complexes were further characterized by molecular modeling. The scattering profiles computed by the program CRYSOL [37] from the crystal structure of the Mpo dimer and from the atomic model of Lf with appropriately added sugars provided good fits to the corresponding experimental data (**Table 2**). These structures were used as rigid bodies in the modeling of the ternary complex by the program SASREF [38] together with the atomic model of Cp with three carbohydrates attached to the appropriate sites of the Cp molecule. The overall shape of the binary Mpo-Cp complex did not significantly change upon the ternary complex formation as suggested by *ab initio* modeling. Therefore, Mpo and Cp subunits were fixed in the rigid body modeling procedure to their conformation in the crystal structure of the binary complex Cp-Mpo. Given the obtained agreement of the Cp-Lf-Mpo with P2 symmetry, the two Lf subunits and two sugar triplets associated with two Cp molecules were translated and rotated symmetrically to optimize the fits to the experimental data. In total, three scattering curves (i.e. from Cp-Lf-Mpo, Cp-Lf and Cp) were simultaneously fitted by a single rigid body model assuming the same arrangement of Cp and Lf in the binary and the ternary complexes. While fitting the Cp data, only the positions of the three sugars with respect to one Cp monomer were adjusted. The fit to the Cp-Lf curve was computed from one instance of the glycosylated binary complex (the other one had the same conformation due to the symmetry) and all the rigid bodies contributed to the scattering of the ternary complex. The biochemically predicted interface between the C-terminal portion of Cp and the N-lobe of Lf was maintained using the appropriate contacts restraints. Multiple independent SASREF runs produced similar results that yielded good fits to all the data (**Table 2**) and the typical model shown in **Figure 4B** agreed well with *ab initio* shapes of both the ternary complex and Cp-Lf. The rigid body model further confirmed the peripheral location of the Lf subunits, which, notably, did not contact the Mpo dimer. The interfaces of Cp-Mpo and of Cp-Lf were therefore not concurrent and the assumption on the same organization of the ternary complex and the binary ones was consistent with the *a posteriori* evidence. The SAXS-derived model allowed for further analysis of the interparticle interfaces in the complex. Interestingly, both lobes of Lf (i.e. N1-domain of N-lobe, C1 and C2 domains of C-lobe) interacted with Cp (domains 1 and 6) (**Figure 4C**), although in the SAXS rigid body modeling procedure the contacts were only required for the N-lobe. The most pronounced contact area between Lf and Cp was clearly in the N1-domain of Lf. The region involving the C1-domain (the loop between the two last alpha-helices) interacted with domain 1 of Cp (**Figure 4C**). The interaction of Lf with Cp *via* the sugar chains seemed to be rather plausible. The N2-domain of Lf is located near the domain 1 of Cp in which a sugar was attached to Asn119. Similarly, Cp may interact with the Lf-C2 mainly *via* the sugar chain (**Figure 4C**), and the carbohydrate chain attached to Asn479 of Lf probably took part in the interaction with domain 1of Cp (**Figure 4C**).

### Gel-filtration of Complexes Cp with Lf and Mpo

The stoichiometry of Cp-Mpo and Cp-Lf-Mpo complexes obtained in the present work contradicted the previous gel-filtration results [18]. To clarify this point, we repeated the experiments using considerably increased amounts of protein in the samples. The complex formation upon mixing Cp with Lf and/or Mpo was confirmed by the elution of these proteins as a single symmetric peak. In the previous measurements, the molecular masses of the Cp–Lf, Cp–Mpo, and Cp–Lf–Mpo complexes were estimated as 215±5, 280±6, and 350±5 kDa, respectively, and these results were interpreted as complexes containing equimolar amounts of the individual proteins. In the present gel filtration experiments, the spectral properties of the eluted complexes were also analyzed. The specific ratios *A_280_/A_430_* and *A_280_/A_610_* obtained for the complex Cp and Mpo (see **Table 3**) were indicative of the stoichiometry 2Cp:1 Mpo. The stoichiometry of the ternary complex revealed the spectral ratios consistent with the 2Cp:2Lf:1Mpo stoichiometry. Therefore, the gel-filtration and the spectral data for Cp-Mpo and Cp-Lf-Mpo suggested different complex stoichiometries, the latter method confirming the stoichiometries obtained from the structural data. For the interactions of Cp with Lf, no discrepancy between the gel-filtration and the spectral data was observed, both methods confirmed the stoichiometry of 1Cp:1Lf.

**Table 3:** Spectral properties for protein and its mixtures.

Protein(s)	*A_280_/A_610_*	*A_280_/A_430_*
**Cp**	22	>100
**Mpo**	14.4	1.25
**Theoretical values**		
**1Cp:1Lf**	35	>100
**1Cp:2Lf**	47.8	>100
**1Cp:1Mpo**	14.8	2.35
**2Cp:1Mpo**	16.5	3.45
**1Cp:1Lf:1Mpo**	18.7	3.0
**2Cp:2Lf:1Mpo**	22.6	4.7
**Actual measurements**		
**Cp+Lf**	35.1±0.2	>100
**Cp+Mpo**	16.3±0.2	3.4±0.1
**Cp+Lf+Mpo**	22.4±0.2	4.6±0.1

## Discussion

The structure of the binary complex Cp-Mpo was solved crystallographically and the low resolution models of the ternary complex Cp-Lf-Mpo and of its binary component, Cp-Lf, were reconstructed using X-ray scattering in solution. The SAXS model of the ternary complex is compatible with the crystal structure of the binary complex Cp-Mpo, indicating that the latter complex is likely to preserve its structure in solution. Despite its 4.7Å resolution, the crystallographic structure of the Cp-Mpo complex provides useful insights into the complex formation. The stoichiometry of 2Cp:1Mpo observed in the crystal is in agreement with the SAXS data. Such stoichiometry of the complex both in the crystal and in solution accounts for the dimeric structure of Mpo and correlates well with the enzymatic activity data. Indeed, Mpo was shown to enhance the oxidase activity of Cp towards *p*-PD at molar ratio of 1Mpo:2Cp. Further increase of the amount of Mpo did not change the activity of Cp [20]. An inconsistency was observed with the previous gel filtration results on the ternary complex Cp-Lf-Mpo [18], which prompted us to perform extensive chromatography experiments, in which spectral ratios of proteins eluted from the column were also assayed. These gel filtration experiments yielded the stoichiometry 2Cp:1Mpo and 2Cp-2Lf-1Mpo in full agreement with the structural studies and in line with our previous suggestion based upon the results of photon correlation spectroscopy [35]. The binary complex Cp-Lf displayed the 1∶1 ratio as reported before [18]. The presence of Mpo in a complex caused retardation of the latter, since Mpo interacted with the resin in course of gel filtration. Hence, the elution volume obtained seems to correspond to the 1∶1:1 stoichiometry.

The structure of the Cp-Mpo complex correlates with the earlier reported alterations of spectral properties of both proteins due to complex formation [20]. Indeed, we observed changes in the absorption spectrum of Mpo upon its interaction with Cp. Adding Cp to Mpo solution caused a shift of the Soret peak maximum (heme-provided spectrum element) from 430 to 427 nm, which could not be explained by a summation of the individual spectra of Cp and Mpo. Differences in absorption maxima for heme-containing proteins are correlated with the specific heme configurations [39]. For instance, lactoperoxidase and horseradish peroxidase are characterized by an intense Soret peak at 413 and 403 nm, respectively. While heme in horseradish peroxidase is a planar structure, it is slightly bent in lactoperoxidase and even more so in Mpo. It seems likely that the interaction with Cp changes the heme conformation in Mpo, which, in turn, may alter the functional activity of Mpo as described before [20].

Cp appears to influence not only the heme conformation, but also the enzymatic activity of Mpo *via* intermolecular contacts. The modeled loop between domains 5 and 6 of Cp (885–892) is expected to contact Mpo in the vicinity of the heme pocket (**Figure 2A,B**). The position of this loop is likely to change upon binding to Mpo as compared to the unbound ceruloplasmin. The superposition of our earlier 2.6 Å Cp structure [30] with the present Cp-Mpo structure shows that the conformation of the loop is indeed changed, probably to avoid steric clashes (**Figure S4**).

The interaction of the interdomain loop of Cp (a.a. 885–892) with the area of the Mpo heme pocket is likely to create a steric barrier precluding the binding of aromatic substrates, and this leads to an inhibitory effect of Cp. Such a mechanism is in good agreement with our previous results showing that Cp did not inhibit the oxidation of small aromatic substrates (guaiacol and orcinol) in contrast to larger ones (*o*-DA, 4-chloro-1-naphtol, tetramethylbenzidine, and ABTS) [20]. The larger the substrate, the stronger the steric barrier and, hence, the more pronounced is the inhibitory effect of Cp upon the peroxidase reaction catalyzed by Mpo. The chlorinating activity of Mpo was also suppressed by Cp [40], [20], and the inhibitory effect can be explained with similar arguments taking into account competition between Cp and Cl^−^ ion (A. S., unpublished data).

In spite of different mechanisms of the two Mpo activities (peroxidation and chlorination), suppression of each of these required that Cp had not been proteolyzed [20]. Accordingly, the heme pocket of Mpo in the Cp-Mpo structure contacts the peptide loop linking domains 5 and 6 in Cp (a.a. 885–893), and this stretch is extremely vulnerable to serine proteinases. It seems likely that the integrity of the interdomain loop in Cp is crucial for its ability to affect Mpo activities, although splitting of the former did not preclude from the complex formation [18].

When bound to an intact Cp molecule, Mpo makes the vulnerable interdomain loop inaccessible for proteinases, which protects Cp against the attack of trypsin, elastase and plasmin, preventing the cleavage of the Cp molecule between domains 5 and 6. Proteolytic cleavage of peptide bonds in Cp beyond the region of protein-protein interaction was also inhibited when it formed a complex with Mpo. This may be explained by a trigger effect: the proteinases are known to hydrolyze peptide bonds in Cp in a certain order, therefore, unless the first one is cleaved, splitting of other bonds does not occur [20].

The proteolytic fragments of Cp that initially do not dissociate and mimic the intact molecule are able to perform as a potent oxidase. The gradual disjunction of the fragments diminish, and ultimately interrupts the electron transfer between the copper ions of the catalytic center in Cp. Copper ions acting independently are likely to acquire prooxidant features observed in the cleaved Cp, in contrast to the intact protein [41]. Thus, Cp-Mpo interaction favors the antioxidant activity of Cp.

When the loop between domains 5 and 6 (a.a. 885–893) of Cp is cut by serine proteinases the β-sheet elements still hold the molecule together. The structure of Cp contains five disulfide bridges stabilizing the external loops between the β-strands. The sequence alignment of five homologous proteins i.e. human and rat Cp, hephaestin, coagulation factors V and VIII shows that these disulfide bridges are highly conservative in all these proteins (**Figures S5, S6**). Taking into account the importance of the external loops for copper incorporation into Cp [42], the evolutionary conservation of disulphide bridges does not seem accidental. The S-S bridge between Cys881 and Cys885 is not the only factor that stabilizes the interdomain loop 885–893, but the end of this loop is additionally stabilized *via* H-bonds of Arg893 with Asp959 based on the our Cp structure. It is the H-bond that orients Asp959 towards the main chain oxygen (**Figures S6E,F**). This residue is semi-conservative in all five proteins and is found at a position always occupied by a negatively charged residue. The H-bond network links two different *β*-sheets, but also restricts the loop end movements in case of proteolysis that results in cleavage of the bond next to Arg893.

We also observed a modification of the functional activity of Cp upon formation of its complex with Mpo, and another Cp-Mpo contact site seems to be important for this modification. Amino acids in domain 4 of Cp localized in close proximity to the *p*-PD-binding site are involved in the contact. This binding site in the Cp molecule includes amino acid residues His667 and Trp669 [34] and also Ser622, as demonstrated in the present work. The likely mechanism underlying the activation by Mpo of *p*-PD oxidation catalyzed by Cp includes participation of Arg185 in Mpo in substrate binding and electron transfer (*via* M668 and the cupredoxin domain) to the mononuclear type I copper. No modifications of Cp oxidase activity towards other substrates (Fe^2+^, *o*-DA, dihydroxyphenylalanine) were observed upon formation of the complex Cp-Mpo [20], [43]. Mpo is not likely to intervene in the substrate binding or electron transfer in Cp. The binding sites for other substrates from the group of synthetic aromatic amines were not localized until now but these substrates do not appear to attach to Cp in the vicinity of the *p*-PD binding site.

The previous SAXS study evidenced the 1∶1 stoichiometry of the Cp-Lf complex and two possible models were constructed with the C-terminal part of Cp interacting either with the N-lobe or with the C-lobe of Lf [21]. The SAXS data taken alone gave no possibility to discriminate between the involvement of either the N- or C-terminal lobe of Lf, yet the former model seemed to be more plausible from the biochemical point of view.

The low resolution SAXS model of the Cp-Lf complex obtained in the present work demonstrates that both lobes of Lf are involved in the interaction with Cp, and this model agrees with other biochemical and biophysical evidence [35].

The extensive contact area in the N-lobe of Lf is not surprising given that this domain is cationic and, therefore, is likely to interact with the negatively charged domains of its partners. It has been noted earlier that residues Arg2–5, Arg25, Arg28, Lys29 and Arg31 constitute a high affinity region for binding a variety of negatively charged molecules, including DNA and lipopolysaccharides [44]. Recently, the crystal structure of Lf in complex with pneumococcal surface protein A showed the specificity of the N1-domain for binding [45]. Using different competitive agents, which displaced Cp from the Cp-Lf complex (DNA, lipopolysaccharides, heparin, peptides), we demonstrated that the N-lobe of Lf is essential for this interaction [14], [17], which is further confirmed by the present SAXS model.

Two peptides with high affinity to Lf- and Mpo-Sepharose were isolated from spontaneously proteolyzed Cp (residues 50–109 and 929–1012), and these peptides may be important for the interactions of Cp both with Lf and with Mpo [18]. These residue stretches provide ligands for type I Cu^2+^ in domain 6 and for coppers of the trinuclear cluster, crucial for oxidase activity of Cp. In the structural model proposed here these peptides are located in close proximity to the active center.

Our structural models can be directly correlated with the functional properties of Cp in the Cp-Lf complex. We described the modification of Cp oxidative features by Lf [15]. When added to Cp in equimolar ratio, Lf promotes Fe^2+^ and *o*-DA binding to Cp, impeded DOPA binding, and had no effect on the *p*-PD affinity towards Cp. In the SAXS model, Lf interacts mostly with domain 6 of Cp (**Figure 3C**), close to type I copper ion and in proximity to the Fe^2+^ and DOPA binding sites, so it seems likely that Lf can influence the affinity of Cp towards these substrates. When interacting with Cp in the vicinity of the DOPA-binding site, Lf forms a steric barrier for this substrate. In addition, the geometry of the proximal Fe^2+^- binding site is likely to be modified upon the complex formation, which yields an increased affinity towards the substrate. We find that Lf influences not only Cp affinity towards substrates but also the maximal rate of the oxidation. Probably, the complex formation modifies the active center of Cp, enhancing ferroxidase reaction but also inhibiting *o*-DA, *p*-PD and DOPA oxidation. Since the excessive Lf interacting with the 1Cp:1Lf complex diminished the affinity of Cp towards *p*-PD, while V_max_ of substrate oxidation remained unaltered, we propose the existence of a low-affinity Lf-binding site in Cp, probably located in domain 4. Lf binding to the low-affinity site results in formation of a 1Cp:2Lf complex. Under such conditions the maximum rate of DOPA and *o*-DA oxidation was reduced, but ferroxidase activity of Cp remained unaltered as compared to the complex 1Cp:1Lf. A second low-affinity binding site of Lf was also described [46]. Although, only the 1∶1 species was found *in vivo* [14], [46]. We did not observe the second binding site as no excessive amounts of Lf were added to Cp in our experiments.

The glycosylation site Asn479, being conserved in human and bovine Lf, is probably responsible for the regulation of the E2 protein of HCV binding activity of Lf and participates in protein-protein interactions [47]. In our model, the carbohydrate chain attached to Asn479 of Lf –C2 is located in the vicinity of domain 1of Cp. It is therefore conceivable that this carbohydrate chain plays some role in complex formation. Lf possesses a large number of specific features, and we did not check whether all of them were altered when the Cp-Lf complex was formed, but rather focused our studies on the most evident properties of the copper enzyme. As we observed, Cp does not preclude, but rather stimulates iron incorporation into apo-Lf [15]. This function must be facilitated by formation of complexes between Cp and Lf. Along with the data on structural interactions of these proteins, there are indications of their physiological synergism as the genes of Cp and Lf are activated during the mammary gland involution and are likely to minimize the oxidative stress of this process [48].

When released from neutrophil granules, Mpo (pI 9–10) and Lf (pI 8–9), definitely cationic proteins, are unlikely to exist in plasma on their own. It seems natural that they act in the bloodstream mainly in complexes with other proteins, which makes the studies of the complexes of Lf and Mpo with Cp highly physiologically relevant. A relatively high affinity of Cp to Mpo and the 250 molar excess of Cp in plasma with respect to all the neutrophilic Mpo suggest that Cp is one of the most likely candidates for the partnership with Mpo. Interactions of Cp with Lf and Mpo can be mediated *via* sugar chains and can affect their clearance from blood circulation [14], [16].

As a member of the transferrin family, Lf can incorporate ferric ions produced during ferroxidase reaction catalyzed by Cp [15]. The progress of inflammation is accompanied by tissue necrosis causing an increment to ferrous iron pool. Brought about by respiratory burst, the active oxygen species (namely hydrogen peroxide, hydroxyl radical, superoxide anion radical) may react with pro-oxidant ions and facilitate the oxidative injuries. Therefore, oxidation of pro-oxidant ferrous ions catalyzed by Cp should reduce the oxidative stress. When oxidized by Cp, a chelating agent should secure the iron ions.

In view of generally low pH in an inflammation focus, iron-binding properties of transferrin are impaired there, and probably Lf will take up ferric ions. Upon interaction of Lf with Cp, the ferroxidase activity of the latter is enhanced, which facilitates capturing iron ions by Lf participating in the complex. Efficient sequestration of iron by the complex and its subsequent clearance from plasma can result in a decrease of the plasma iron concentration. Thus the Cp-Lf complex that we found in the blood of patients with inflammatory diseases might be a mechanism of an organism’s protection against the neutrophilic respiratory burst in an inflammation focus [16]. Interaction of Lf with Cp may regulate the generation of OH˙ from hydrogen peroxide in the foci of inflammation and protect the adjacent tissues [49].

It was shown previously that only intact Cp was able to catalyze the incorporation of iron into ferritin. Besides, it prevented an alteration of ferritin during the process, despite that the rate of ferroxidase reaction that it catalyzed was only 1.7 times higher than that for the protested form of Cp [9]. One should not exclude that incorporation of iron into lactoferrin can also be affected by proteolysis of Cp. On account that Mpo protects Cp against proteolysis the formation of the ternary complex Cp-Lf-Mpo possibly favors iron incorporation into apo-Lf.

Interaction with Cp inhibited peroxidase activity of Mpo under physiological conditions [10], [19], [20] and prevented HOCl production by Mpo [5], [39]. HOCl can damage all classes of biopolymers and is a strong antibacterial agent playing an important role in cell defense. Interaction of Cp with Lf and Mpo may be a part of a regulatory mechanism for neutralizing or modulating biological activity of potentially toxic proteins of neutrophils, once those appear in the bloodstream. Synergism of bactericidal effects of Mpo and Lf was demonstrated [50]. Our SAXS model reveals the absence of interaction between Mpo and Lf in the ternary complex, so Cp can serve as a mediator of protein interactions in the complex architecture and in the connection of the complex components to the putative downstream targets of the complex activity.

**Figure 5:**
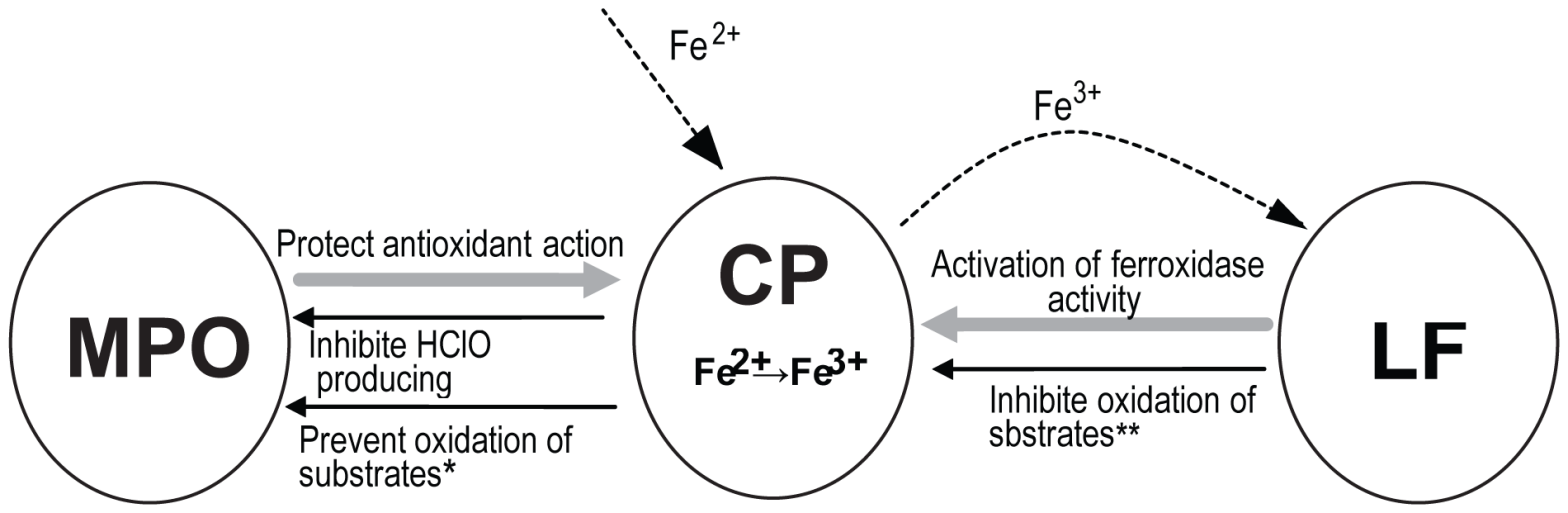
Scheme of influence of Cp, Mpo, Lf on functions of each other due to interactions. Inhibition is shown by thin black arrows, activation or protection of function is shown by bold grey arrows. *Substrates of Mpo: o-DA, 4-chloro-1-naphol, tetramethylbenzidine, ABTS; **Substrates of Cp: *o*-DA, *p*-PD, DOPA.

Our structural results support the notion of an intricate system of alteration in the functional properties of individual proteins due to complex formation (**Figure 5**). Here, Cp inhibits Mpo-induced HClO production and prevents oxidation of Mpo substrates. Mpo protects the antioxidant properties of Cp by isolation of the sensitive loop of Cp from proteases. This protection is important for the incorporation of Fe^3+^ into Lf, activating the ferroxidase activity of Cp and precluding the oxidation of Cp's substrates. Occurrence of such complexes in inflammation foci is likely to extenuate the results of respiratory burst in neutrophils or to prevent the origination of Cp derivatives (proteolytic fragments) possessing pro-oxidant activity.

Our data show that a copper protein and two iron proteins, all possessing a variety of functions, are able to form molecular assemblies with complex structure. The ability of these secretory proteins to associate, which results in modification of their functions, does not seem accidental. In fact, our previous data indicate that complexes Cp-Lf, Cp-Mpo and Cp-Lf-Mpo are not infrequently found in biological fluids of patients with inflammation [16]. Structural data presented here elucidate the mechanism by which the very formation of a complex of Cp with its partner(s) enforces the antioxidant activity of the copper enzyme.

## Materials and Methods

### Ethics Statement

Plasma, leukocytes and breast milk samples for the purification of proteins were obtained with written informed consent of healthy donors. The Committee on biomedical ethics of the Institute for Experimental Medicine approved this study.

### Isolation and Purification of Proteins

Cp was obtained by affinity chromatography on protamine-Sepharose. Preparation had *A_610_/A_280_*>0.045 and for more than 95% consisted of non-fragmented protein [20].

Mpo was isolated from human leukocytes as was described before [18].

Lf was purified from breast milk by ion-exchange chromatography on CM-Sephadex and gel-filtration on Sephadex G-100 [14].

### Gel-filtration of Proteins

Gel-filtration of proteins was performed on a column (1×50 cm) packed with Toyopearl HW-55 fine. Proteins were eluted with 0.15 M NaCl in 10 mM Tris-HCl, pH 7.4, at a flow rate of 0.5 ml/min. Samples having volume 100 µl contained: a) mixture of Cp (5 mg) and Lf (3 mg); b) Cp (5 mg) and Mpo (3 mg); c) Cp (5 mg), Lf (6 mg), and Mpo (3 mg). Ferritin, catalase, Mpo, Cp, aldolase, and Lf (2 mg per 100 µl of solution) were used as molecular mass markers. Based on the results of three measurements, a calibration graph was plotted and the molecular mass of the complexes was calculated. Spectral properties of fractions eluted in single peaks (for complexes) were measured to check the relative amounts of proteins there. Since Cp has absorption maximum at 610 nm, and Mpo – at 430 nm, we estimated specific spectral ratios (*A_280_/A_610_*, *A_280_/A_430_*) and compared these with calculated theoretical values.

### Peroxidase Activity of Mpo

To study an effect of synthetic peptide RPYLKVFNPR (corresponding to the fragment of Cp sequence 883–892) on oxidation of ABTS by Mpo [20] we used a mixture of 10 nM Mpo, 0.1 mM H_2_O_2_ and 1 mM ABTS in 0.1 M sodium-acetate buffer, pH 5.6 and various amounts of the peptide (50–800 nM). The reaction was triggered by adding H_2_O_2_ to the mixture and its rate at room temperature was measured as Δ*A*_414_/min.

### X-ray Analysis of Cp-Mpo Complex and Cp with Free Labile Sites

The complex was obtained pre-mixed from Cp and Mpo (1∶1) before crystallization. Hexagonal crystal of the Cp-Mpo complex was grown using the crystallization robot Hydra-II and MPD Suite Screening (Qiagen). The crystal referred to the space group P6_5_22 and had the unit cell parameters: a = b = 106.3 Å c = 836.5 Å. Diffraction data set was collected up to 4.7Å resolution at the BW6 beamline, DESY (Hamburg, Germany). The data were processed using DENZO and SCALPACK software [51]. The structure was solved using MOLREP [52]. Structures of Cp **(4enz**) and of one of Mpo monomers (**1cxp**) were used as models. Rigid body refinement was performed by REFMAC5 [53] with overall B-factor.

Structure of Cp with free labile sites at 2.6 Å was refined by REFMAC5 using TLS. This allowed improved refinement statistics compared to previous reports [30].

Data collection and refinement statistics are summarized in **Table 1**. Accessible surface area was calculated by Areaimol program from the CCP4 package.

### SAXS Measurements and Data Processing

SAXS measurements were performed using the X33 beamline of EMBL, DESY (Hamburg, Germany). All samples of Cp-Lf, Cp-Lf-Mpo and individual proteins were measured at several solute concentrations ranging from 1 to 5 mg/ml. All the data sets were measured using a MAR345 Image plate (Norderstedt, Germany), except for Cp-Lf-Mpo, which was measured on a PILATUS-1M pixel detector (Baden, Switzerland). At a sample-detector distance of 2.7 m, the range of momentum transfer 0.12< *s* <5 nm^−1^ was covered (*s = 4π sin(θ)/λ* where *2θ* is the scattering angle and λ = 0.15 nm is the X-ray wavelength). The data were processed using standard procedures in the program package PRIMUS [54]. Multiple scattering patterns belonging to the same construct were extrapolated to infinite dilution to remove interpatricle interference effects at small angles and merged with the highest measured concentration at higher angles. The forward scattering, *I(0),* and the radii of gyration, *R_g_* were evaluated using the Guinier approximation [55], assuming that at very small angles the intensity is represented as *I(s) = I(0) exp(–(sR_g_)^2^/3)*. The requirement that *s<1.3/R_g_* required for the Guinier fitting was fulfilled for all constructs by using the automated program AutoRg [56]; all Guinier plots in the insert of **Figure 4A** obey this condition. The maximum dimensions *D_max_* was computed using the indirect transform package GNOM [57], which also provides the distance distribution function *p(r)*. The increase in the molecular mass upon complex formation was verified by the analysis of the excluded particle volume *V_p_* computed from the scattering data using the Porod invariant.

Low resolution shape analysis of the solutes was performed using the *ab initio* bead modeling program DAMMIN, which employs simulated annealing (SA) to build a compact interconnected configuration of beads inside a spherical search volume with the diameter *D_max_* that fits the experimental data *I_exp_(s)* by minimizing the discrepancy

(1)where *N* is the number of experimental points, *c* is a scaling factor and *I_calc_(s)* and σ *(s_j_)* are the calculated intensity and the experimental error at the momentum transfer *s_j_*, respectively. The shape reconstructions of ternary complex were made assuming no symmetry and also with P2 symmetry constraints.

Molecular modeling against SAXS data was performed by the program SASREF based on the atomic models of individual subunits (PDB entries **4enz**, **1lfi**, **1dnu** for Cp, Lf and Mpo, respectively). SASREF uses SA to position the rigid bodies with respect to each other, so that an interconnected assembly without steric clashes is formed, while minimizing the discrepancy between the SAXS experimental data and the scattering profiles computed from the corresponding sets of subunits. The scattering calculation is based on the pre-computed partial amplitudes of the subunits in the reference positions and orientations provided by CRYSOL [37], which either predicts theoretical scattering patterns or fits the experimental data by adjusting the excluded volume and the contrast of the hydration layer. To account for glycosylations, sugar molecules were introduced as additional rigid bodies anchored to the appropriate residues using contacts restraints having a form of a spring-force potential. The contacts restraints were also used to maintain the contact between the C-terminal part of Cp and the N-lobe of Lf, which has been predicted biochemically [16].

The *ab initio* and rigid body modeling procedures were repeated several times for all proteins and complexes to verify the stability of the solution, and the most typical reconstructions were selected using the programs DAMAVER [58] and SUPCOMB [59].

### Accession Numbers

The PDB accession numbers for the Cp-Mpo and Cp with free labile sites reported in this paper are **4enz** and **4ejx**.

## Supporting Information

Figure S1 **Overall structure organization of Cp and Lf.** Right view is rotated by 90 degrees around horizontal axis. The molecule is represented as cartoon; its six domains are marked in Roman numerals and colored as follows: domain I in light blue, domain II in blue, domain III in pale cyan, domain IV in purple, domain V in dark sky blue and domain VI in cyan. Copper ions are shown as orange spheres. (**B**) Structural organization of Lf. Lf molecule is shown in cartoon representation. Domain 1 of N-lobe is shown in chocolate, domain 2 of N-lobe is shown in red, domain 1 of C-lobe is shown in pink and domain 2 of C-lobe is shown in dark pink. Two inter-lobe helices are shown in grey. Ferrous ions are shown as black spheres.
(TIF)

Figure S2 **Contacts between symmetrical molecules of individual proteins in Cp-Mpo complex.** (**A**) Overall view of contacts between two symmetrical molecules of Mpo which forms a crystallographic dimer. Symmetrical molecule is shown by light green and labeled with *. (**B**) Electron density map 2Fo-Fc at 1σ level for two symmetrical sugar chains is shown. (**C,D**) Contact between Cps molecules related by 2-fold axes. Symmetrical subunit is shown in cyan and labeled with *.
(TIF)

Figure S3 **Phenylenediamine binding site in free labile sites Cp structure.** Phenylenediamine binding site in free labile sites Cp structure, related to Figure 3,D. Residues are shown in stick representation, water molecule are shown in red sphere. H-bonds are shown in dash lines. *2Fo-Fc* electron density map is shown at 1 σ level.
(TIF)

Figure S4 **Possible changing of loop tracing during Cp-Mpo complex formation in order to avoid steric clashes.** Possible changing of loop tracing during Cp-Mpo complex formation in order to avoid steric clashes, related to Discussion. (**A**) Superposition of Cp and Cp-Mpo structure. Proteins are shown in cartoon representation. Free Cp is shown in dark blue, loop 885–892 is shown in yellow; Cp in Cp-Mpo complex is shown in pale cyan, loop 885–892 is shown in dark red. Area of interaction between Cp an Mpo is marked by dashed red oval. (**B**) *2Fo–Fc* electron density map shown at 1 σ level for visible residues of interdomain loop 885–892.
(TIF)

Figure S5 **Sequence alignment of five homology protein fragments: human Cp, rat Cp, human hephaestin, human coagulation factor V and coagulation factor VIII.** Sequence alignment of five homology protein fragments: human Cp, rat Cp, human hephaestin, human coagulation factor V and coagulation factor VIII, related to Discussion. Only fragments containing disulfide bridges are shown. Cysteins are shown in green. Conservative metal-binding residues are shown in red; half-conservative metal-binding residues are shown in magenta. Disulfide bridges are shown by green dash-lines. Numbers of cystein residues are shown in green (Cp numbering).
(TIF)

Figure S6 **Elements of β-sheets stabilization in Cp structure.** Elements of β-sheets stabilization in Cp structure, related to Discussion. (**A–D**) Conservative disulfide bridges in the Cp structure. Cp molecule is shown in cartoon representation, cysteins are shown by sticks. (**E**) Elements stabilizing the beginning and the end of the loop between domains 5 and 6. Disulfide bridge between Cys881 and Cys855 and H-bond network. (**F**) 2Fo–Fc electron density map countered at 1σ level for residues forming H-bond networks in the end of the domain 5-domain 6 loop.
(TIF)
