# Supplementary figures and images for: Ceruloplasmin: Macromolecular Assemblies with Iron-Containing Acute Phase Proteins
Source: PLoS One. 2013 Jul 3;8(7):e67145. doi: 10.1371/journal.pone.0067145 (PMC3700992; doi:10.1371/journal.pone.0067145)

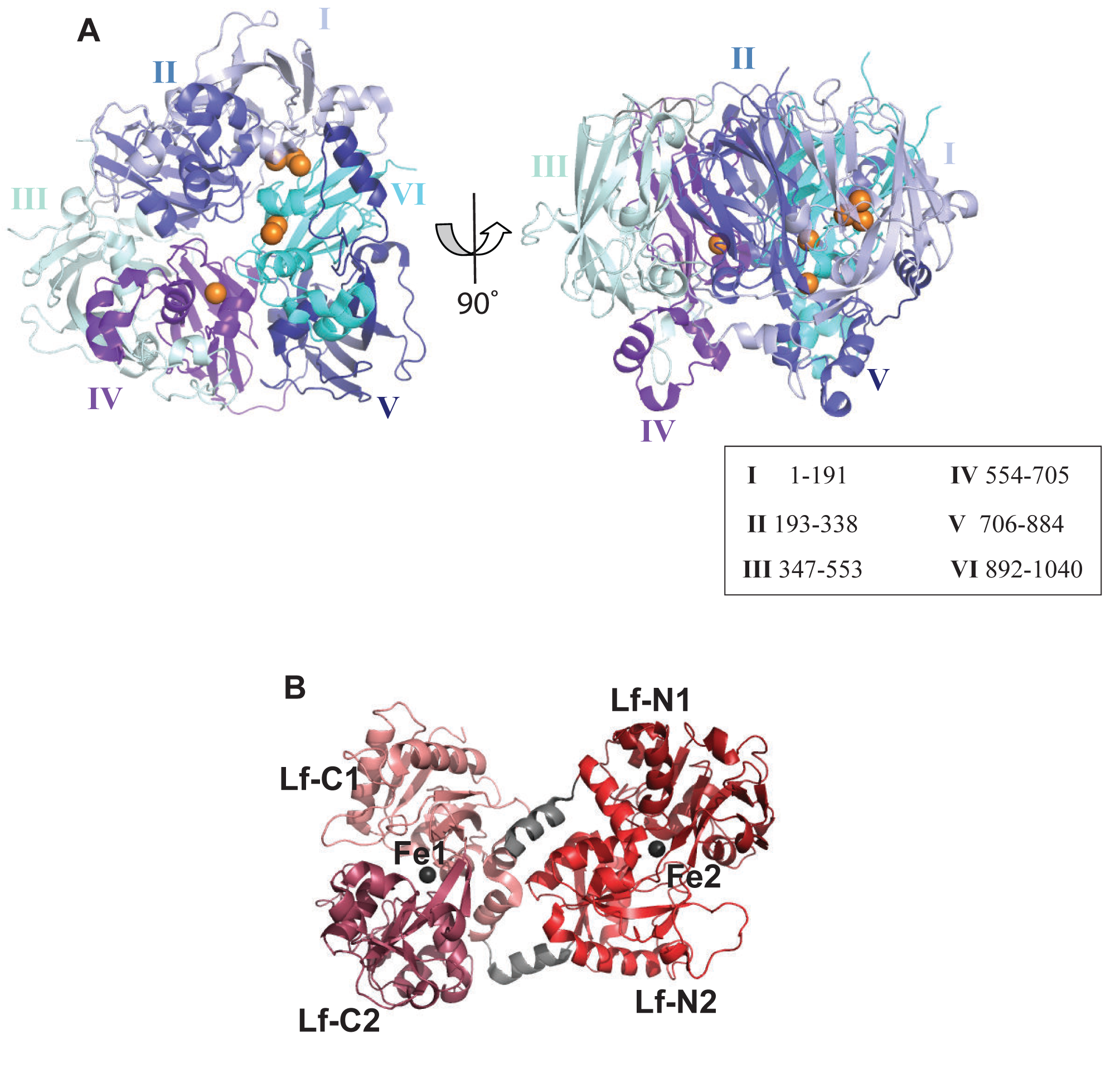

Supplement: Figure S1 — Overall structure organization of Cp and Lf. Right view is rotated by 90 degrees around horizontal axis. The molecule is represented as cartoon; its six domains are marked in Roman numerals and colored as follows: domain I in light blue, domain II in blue, domain III in pale cyan, domain IV in purple, domain V in dark sky blue and domain VI in cyan. Copper ions are shown as orange spheres. (B) Structural organization of Lf. Lf molecule is shown in cartoon representation. Domain 1 of N-lobe is shown in chocolate, domain 2 of N-lobe is shown in red, domain 1 of C-lobe is shown in pink and domain 2 of C-lobe is shown in dark pink. Two inter-lobe helices are shown in grey. Ferrous ions are shown as black spheres. (TIF) [file pone.0067145.s001.tif]

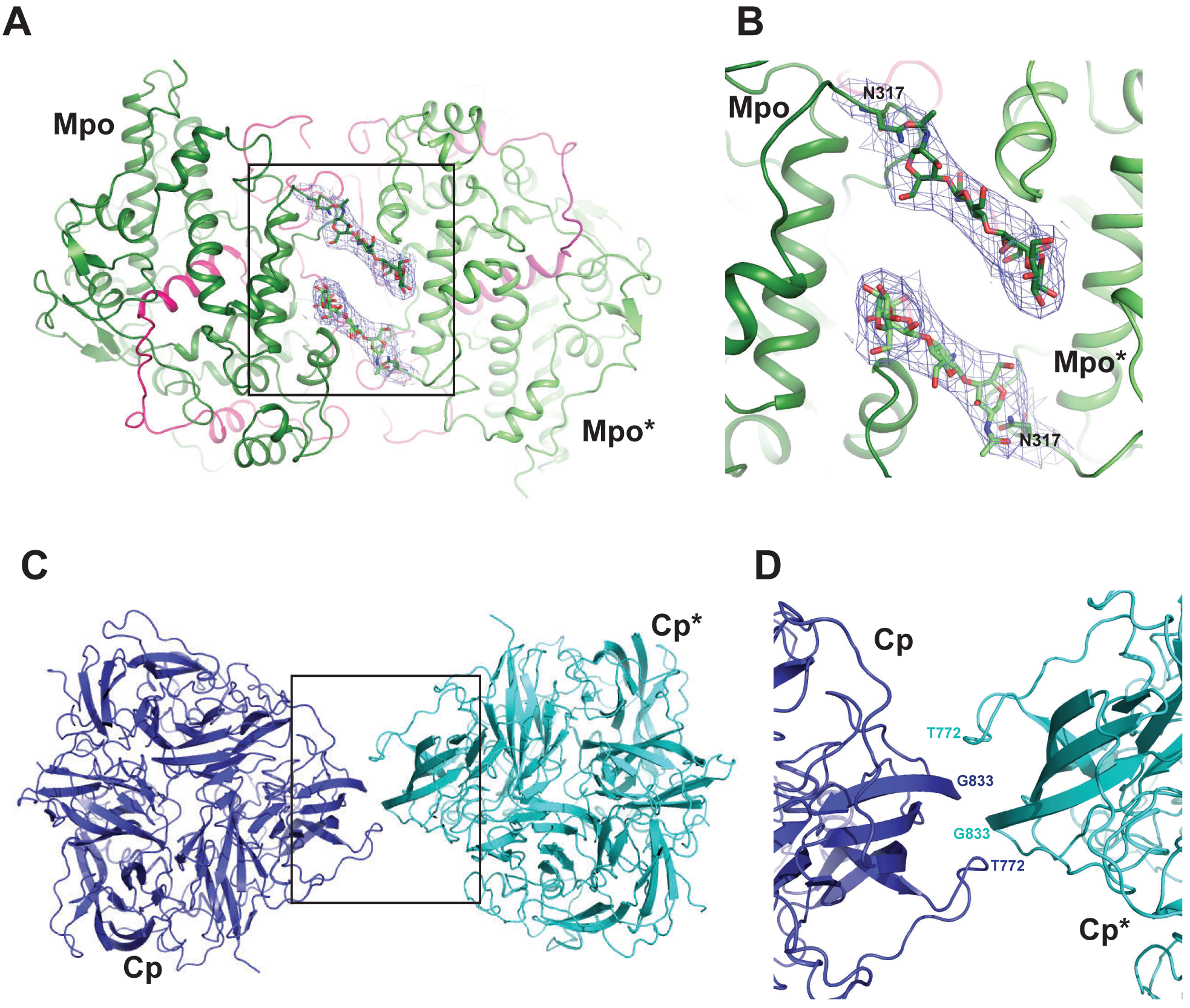

Supplement: Figure S2 — Contacts between symmetrical molecules of individual proteins in Cp-Mpo complex. (A) Overall view of contacts between two symmetrical molecules of Mpo which forms a crystallographic dimer. Symmetrical molecule is shown by light green and labeled with *. (B) Electron density map 2Fo-Fc at 1σ level for two symmetrical sugar chains is shown. (C,D) Contact between Cps molecules related by 2-fold axes. Symmetrical subunit is shown in cyan and labeled with *. (TIF) [file pone.0067145.s002.tif]

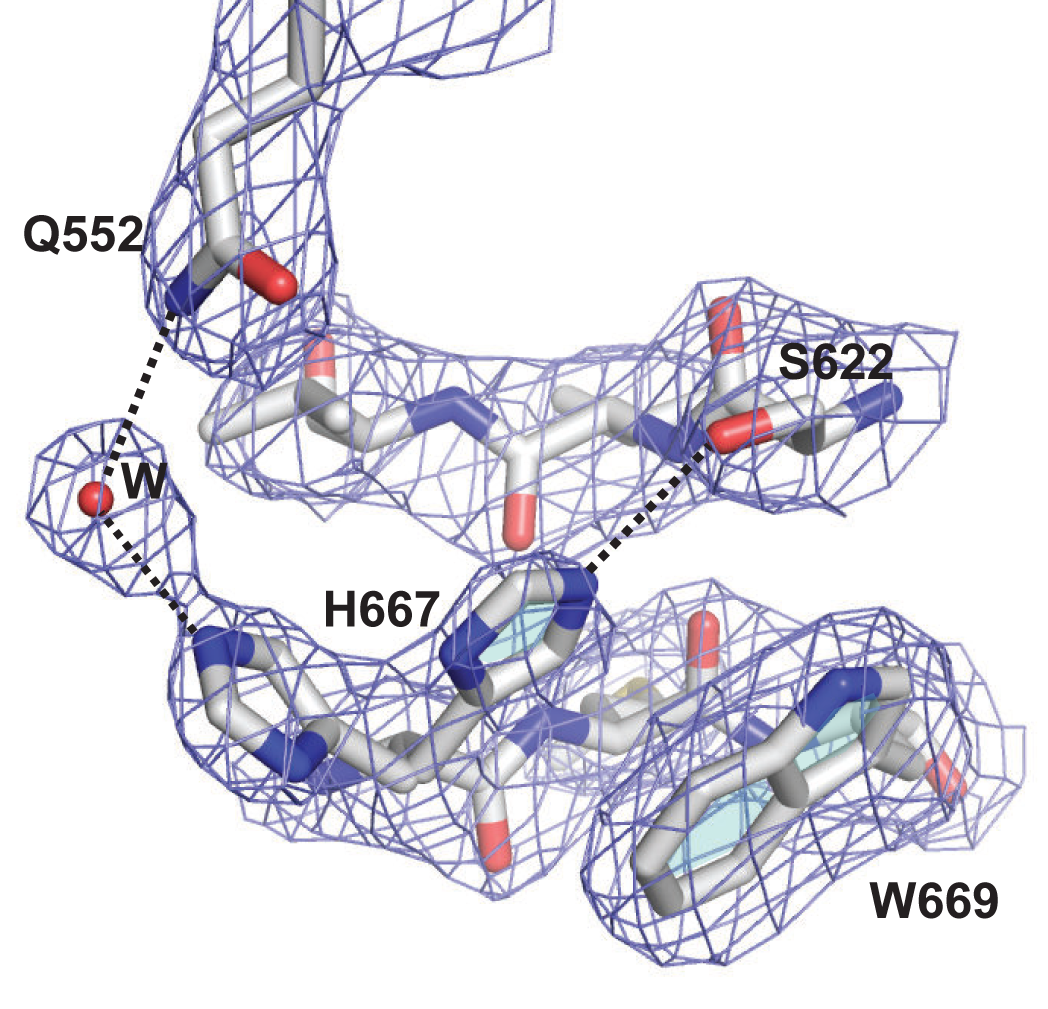

Supplement: Figure S3 — Phenylenediamine binding site in free labile sites Cp structure. Phenylenediamine binding site in free labile sites Cp structure, related to Figure 3,D. Residues are shown in stick representation, water molecule are shown in red sphere. H-bonds are shown in dash lines. 2Fo-Fc electron density map is shown at 1 σ level. (TIF) [file pone.0067145.s003.tif]

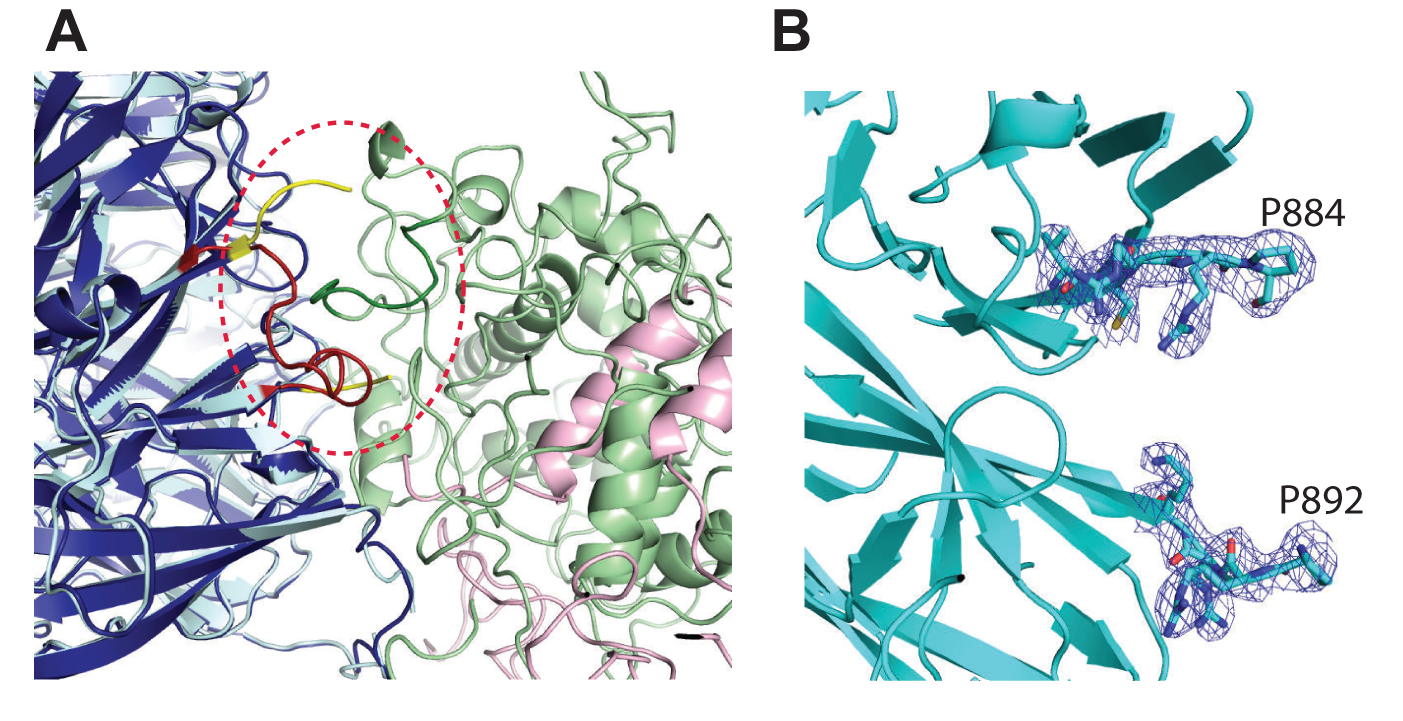

Supplement: Figure S4 — Possible changing of loop tracing during Cp-Mpo complex formation in order to avoid steric clashes. Possible changing of loop tracing during Cp-Mpo complex formation in order to avoid steric clashes, related to Discussion. (A) Superposition of Cp and Cp-Mpo structure. Proteins are shown in cartoon representation. Free Cp is shown in dark blue, loop 885–892 is shown in yellow; Cp in Cp-Mpo complex is shown in pale cyan, loop 885–892 is shown in dark red. Area of interaction between Cp an Mpo is marked by dashed red oval. (B) 2Fo–Fc electron density map shown at 1 σ level for visible residues of interdomain loop 885–892. (TIF) [file pone.0067145.s004.tif]

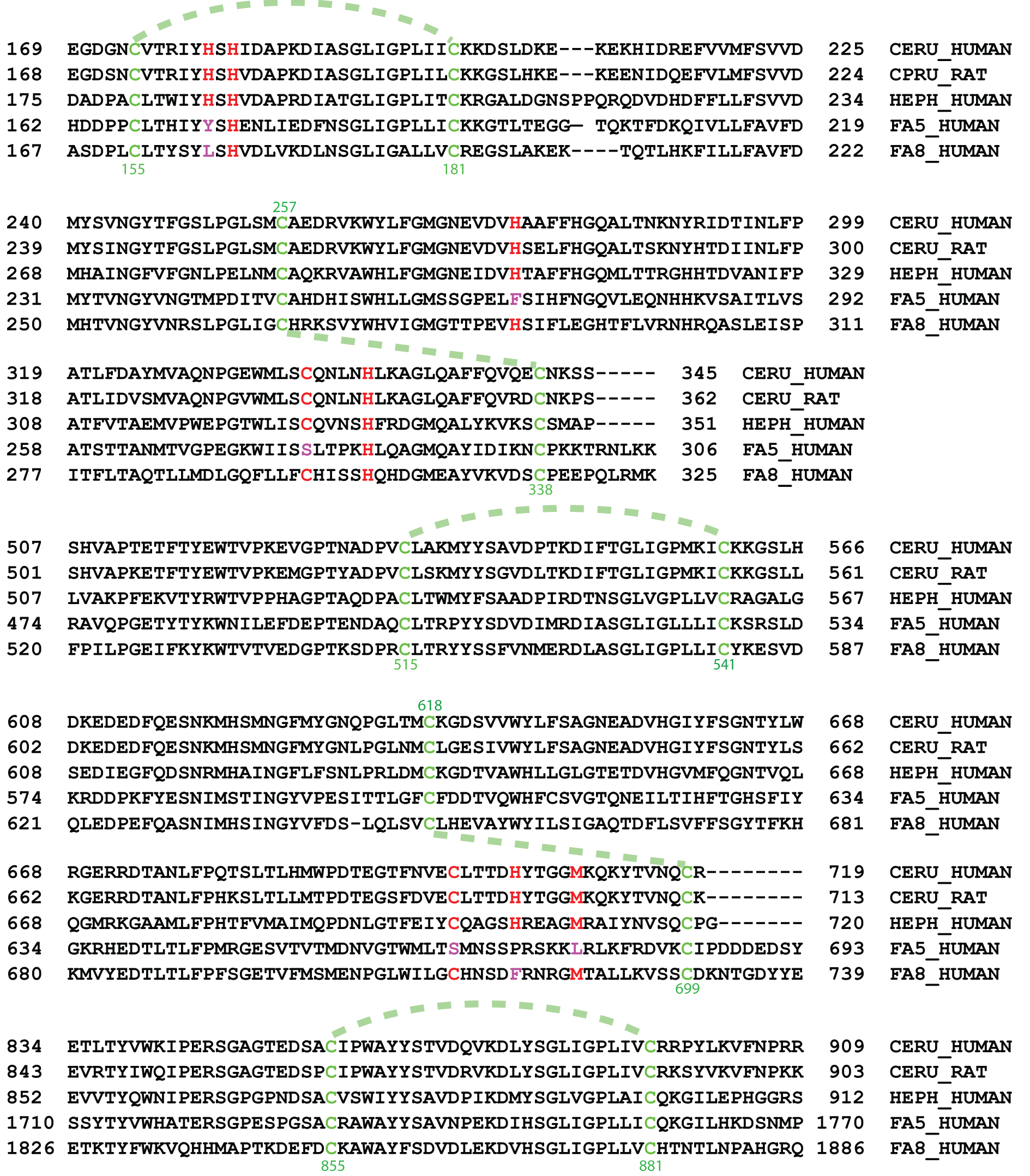

Supplement: Figure S5 — Sequence alignment of five homology protein fragments: human Cp, rat Cp, human hephaestin, human coagulation factor V and coagulation factor VIII. Sequence alignment of five homology protein fragments: human Cp, rat Cp, human hephaestin, human coagulation factor V and coagulation factor VIII, related to Discussion. Only fragments containing disulfide bridges are shown. Cysteins are shown in green. Conservative metal-binding residues are shown in red; half-conservative metal-binding residues are shown in magenta. Disulfide bridges are shown by green dash-lines. Numbers of cystein residues are shown in green (Cp numbering). (TIF) [file pone.0067145.s005.tif]

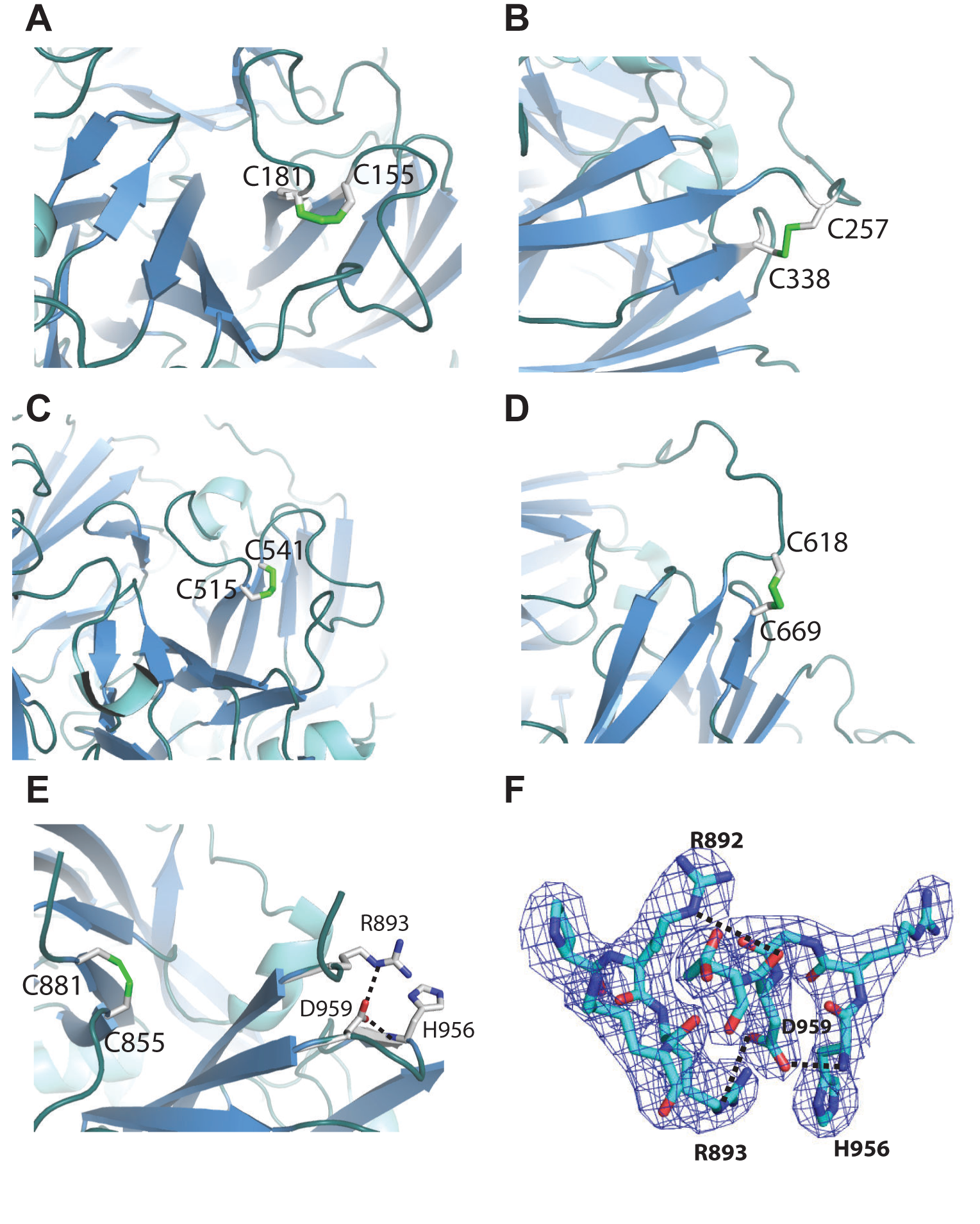

Supplement: Figure S6 — Elements of β-sheets stabilization in Cp structure. Elements of β-sheets stabilization in Cp structure, related to Discussion. (A–D) Conservative disulfide bridges in the Cp structure. Cp molecule is shown in cartoon representation, cysteins are shown by sticks. (E) Elements stabilizing the beginning and the end of the loop between domains 5 and 6. Disulfide bridge between Cys881 and Cys855 and H-bond network. (F) 2Fo–Fc electron density map countered at 1σ level for residues forming H-bond networks in the end of the domain 5-domain 6 loop. (TIF) [file pone.0067145.s006.tif]
